# A Multi-Omics Interpretable Machine Learning Model Reveals Modes of Action of Small Molecules

**DOI:** 10.1038/s41598-020-57691-7

**Published:** 2020-01-22

**Authors:** Natasha L. Patel-Murray, Miriam Adam, Nhan Huynh, Brook T. Wassie, Pamela Milani, Ernest Fraenkel

**Affiliations:** 1 Computational and Systems Biology Graduate Program, Massachusetts Institute of Technology, 77 Massachusetts Avenue, Cambridge, MA 02139 USA; 2 Department of Biological Engineering, Massachusetts Institute of Technology, 77 Massachusetts Avenue, Cambridge, MA 02139 USA; 3 Broad Institute, Cambridge, MA 02139 USA

**Keywords:** Machine learning, Network topology, Target identification

## Abstract

High-throughput screening and gene signature analyses frequently identify lead therapeutic compounds with unknown modes of action (MoAs), and the resulting uncertainties can lead to the failure of clinical trials. We developed an approach for uncovering MoAs through an interpretable machine learning model of transcriptomics, epigenomics, metabolomics, and proteomics. Examining compounds with beneficial effects in models of Huntington’s Disease, we found common MoAs for compounds with unrelated structures, connectivity scores, and binding targets. The approach also predicted highly divergent MoAs for two FDA-approved antihistamines. We experimentally validated these effects, demonstrating that one antihistamine activates autophagy, while the other targets bioenergetics. The use of multiple omics was essential, as some MoAs were virtually undetectable in specific assays. Our approach does not require reference compounds or large databases of experimental data in related systems and thus can be applied to the study of agents with uncharacterized MoAs and to rare or understudied diseases.

## Introduction

Unknown modes of action of drug candidates can lead to unpredicted consequences on effectiveness and safety. Computational methods, such as the analysis of gene signatures, and high-throughput experimental methods have accelerated the discovery of lead compounds that affect a specific target or phenotype^1–3^. However, these advances have not dramatically changed the rate of drug approvals. Between 2000 and 2015, 86% of drug candidates failed to earn FDA approval, with toxicity or a lack of efficacy being common reasons for their clinical trial termination^4,5^. Even compounds identified for binding to a specific target can have complex downstream functional consequences, or modes of action (MoAs)^6^. Understanding the MoAs of compounds remains a crucial challenge in increasing the success rate of clinical trials and drug repurposing efforts^4,6^.

Computational approaches have contributed to the discovery of MoAs. Using the Connectivity Map data, tools like MANTRA can predict MoAs of new compounds based on their gene expression similarity to reference compounds with known MoAs^7^. To combat antibiotic resistance, reference compounds were also used to infer MoAs of uncharacterized antimicrobial compounds by comparing their untargeted metabolomic profiles in bacteria^8^. From human cancer cell lines, basal gene expression signatures were correlated with sensitivity patterns of compounds to identify previously unknown activation mechanisms and compound binding targets^9^. Similarly, gene expression profiles of human lymphoma cells treated with anti-cancer drugs were compared using the gene regulatory network-based DeMAND algorithm to predict novel targets and unexpected similarities between the drugs^10^. However, all of these methods require prior context-specific knowledge, such as data from reference compounds with known MoAs, sensitivity data, or gene-regulatory interactions.

More general approaches to discover MoAs are urgently needed. In the context of late-onset neurodegenerative disorders like Huntington’s Disease (HD), screening efforts focused on protein aggregation, neuronal death, and caspase activation phenotypes have found many compounds that have disease-altering potential, but none have been successful in clinical trials^11^. HD is an autosomal dominant, fatal, neurodegenerative disorder that results in massive striatal neuronal cell death^12^. The disease is caused by a trinucleotide repeat expansion in the huntingtin gene, which encodes an expanded polyglutamine domain in the huntingtin protein^12^. Although the exact function of huntingtin is unclear, it has been shown to interact with many proteins and to be involved in transcription, anti-apoptotic activity, and the trafficking processes of vesicles and organelles^13^. Within brain cells, mutant huntingtin causes transcriptional dysregulation, impaired cytoskeletal motor functions, compromised energy metabolism, and abnormal immune activation^13^.

Over the years, many compounds have been discovered that confer a protective effect in HD model systems^14^. In some cases, direct binding targets are known, but these may not always be in the therapeutic pathway. A study using a small molecule sphingolipid enzyme inhibitor, for example, found a novel MoA related to histone acetylation through the analysis of gene expression and epigenetic profiles in the murine STHdh^Q111^ HD cell model^15^. As all small molecule therapeutics have so far failed to modify HD in clinical trials, understanding the disease-relevant MoAs is critical to guide future therapeutic approaches that could target these pathways with new molecules.

We reasoned that the discovery of MoAs must begin with an unbiased approach. Some compounds may have largely transcriptional effects, while others may primarily impact signaling or metabolism. With improvements in omics technology, it is now possible to systematically assess each of these areas. Technologies such as RNA-Seq, ChIP-Seq, and mass spectrometry provide extensive measurements of gene expression, chromatin accessibility, metabolite expression, protein expression, and post-translational modifications. The integration of these omics data can provide a more comprehensive view of the compounds and allow for discoveries that could be overlooked in the analysis of any individual dataset^16^.

**Figure 1:**
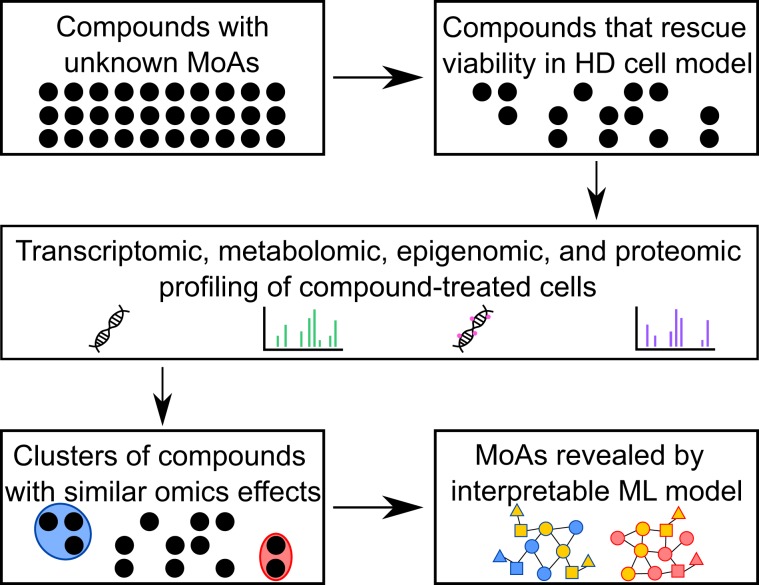
General workflow of study. Compounds with unknown MoAs were found to be protective in HD cells. After multi-omics profiling, groups of protective compounds were shown to cluster together. An interpretable machine learning (ML) model revealed compounds’ MoAs, which were validated experimentally.

To systematically reveal disease-relevant MoAs, we developed a multi-omics machine learning approach (Fig. 1) that does not require context-specific prior knowledge or reference compounds. We used a hierarchical data generation strategy and began with a set of compounds previously reported to alleviate an HD phenotype in at least one HD model system. We filtered the compounds using a viability assay to find those that are protective in the well-established murine striatal STHdh^Q111^ HD cell model. We then profiled compound-treated cells using transcriptomics and untargeted metabolomics. Interestingly, we show that previously unrelated compounds cluster together based on their molecular profiles. For two interesting clusters of compounds, we then gathered proteomic data and epigenomic data.

To reveal the MoAs for these compounds, we applied an interpretable machine learning algorithm. We mapped each type of molecular data to a network of molecular interactions. Network optimization of this large interactome highlights the functional changes induced by the compounds. This approach prioritized two disease-relevant processes, autophagy activation and mitochondrial respiration inhibition, as key MoAs of a subset of these compounds. Through cellular imaging, biochemical, and energetics assays, we confirmed these MoAs in the STHdh^Q111^ murine model. We also demonstrated that the effects on autophagy are reproducible across species and across cell types. This multi-omics approach opens new opportunities for the discovery of existing compounds that may have beneficial effects through unexpected pathways. Equally important, it may provide insight into unrecognized off-target effects on pathways that may contribute to toxicity. Our findings reinforce the importance of unbiased multi-omics approaches in the study of disease and therapeutics.

## Results

### Cell viability assay categorizes compounds by protectiveness

**Figure 2:**
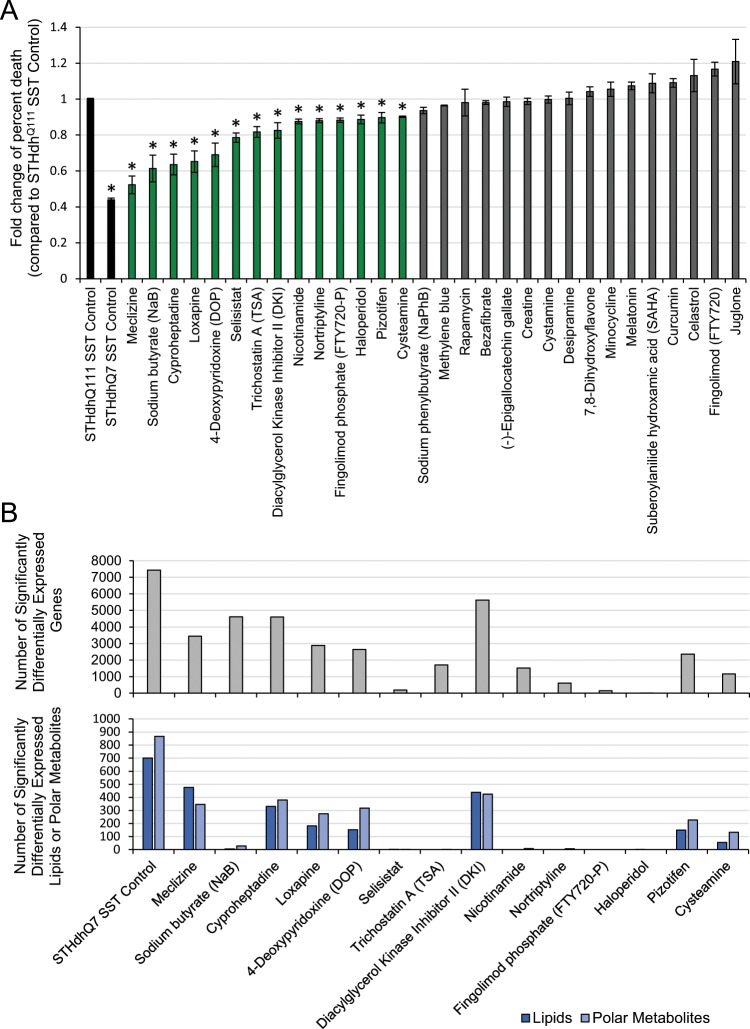
Compounds have diverse effects on viability, gene expression, and metabolite expression in the STHdh^Q111^ cell model. (**A**) Cell viability assay categorizes 14 compounds as protective and 16 as unprotective. Data are represented as mean ± SD. *p-value < 0.001. The black, green, and gray bars indicate controls, protective compounds, and unprotective compounds, respectively. (**B**) The number of transcriptomic and metabolomic changes in compound-treated cells compared to controls.

More than 100 compounds were previously reported to reverse a disease phenotype in at least one HD model system^17^. We examined 30 of these compounds that were commercially available (Table S1) and determined their protectiveness in the well-established STHdh cell culture model of HD. These murine striatal neuronal progenitor cells express the polyglutamine-expanded (STHdh^Q111^) or wild-type (STHdh^Q7^) human huntingtin gene^18^. As has been previously reported, STHdh^Q111^ and STHdh^Q7^ cells differ in their sensitivity to serum deprivation^18^. As a result, we tested the ability of compounds to extend the viability of STHdh^Q111^ cells under these conditions. Of the compounds, 14 were significantly protective (p-value < 0.001) when compared to the STHdh^Q111^ vehicle control (Fig. 2A). The remaining 16 compounds either did not significantly decrease cell death or were toxic to the cells at all tested concentrations.

### Molecular profiles reveal unexpected similarities between compounds

To assess the compounds’ molecular effects on transcription and metabolism, we performed RNA-Seq and untargeted metabolite profiling on STHdh^Q111^ cells treated with the 14 protective compounds and vehicle control, in triplicate. We also included the STHdh^Q7^ vehicle control for comparison. We measured the levels of 18,178 genes, 1,530 untargeted lipids, and 1,805 untargeted polar metabolites in all samples. In most of the compound-treated samples, we found thousands of statistically significant differentially expressed genes (FDR-adjusted p-value < 0.05) compared to the STHdh^Q111^ vehicle control (Fig. 2B). Though some compounds affected several hundred measured metabolites, many of the compounds had little effect on the lipids and polar metabolites (Fig. 2B).

**Figure 3:**
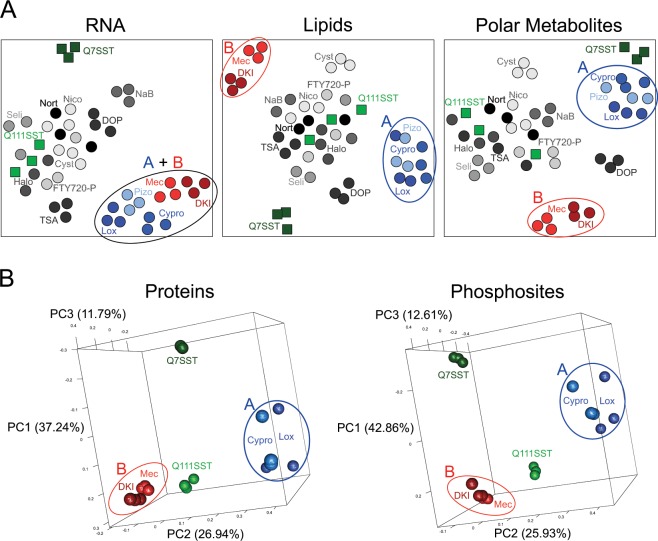
Omics profiles reveal unexpected similarities between compounds. (**A**) Clustering of metabolite profiling data reveals two distinct groups of compounds that are inseparable in the gene expression data, as displayed in t-SNE plots. The blue and red ellipses indicate the Group A and Group B compounds, respectively. Q7SST = STHdh^Q7^ SST control; Q111SST = STHdh^Q111^ SST control; Mec = meclizine; NaB = sodium butyrate; Cypro = cyproheptadine; Lox = loxapine; DOP = 4-deoxypyridoxine; Seli = selisistat; TSA = trichostatin A; DKI = diacylglycerol kinase inhibitor II; Nico = nicotinamide; Nort = nortriptyline; FTY720-P = fingolimod phosphate; Halo = haloperidol; Pizo = pizotifen; Cyst = cysteamine. (**B**) Clustering of proteomic data, as shown in three-dimensional PCA plots. See also Figs S1–S3, Tables S2–S6.

To reveal similarities between the compounds’ profiles, we clustered the RNA, lipid, and polar metabolite data separately (Fig. 3A). In the gene expression data, five compounds reproducibly clustered tightly together in a group distinct from the STHdh^Q111^ vehicle control samples. Although these compounds formed only one distinct group in the gene expression data, they separated into two distinct groups in the metabolite profiling data. Cyproheptadine, loxapine, and pizotifen form Group A and were previously shown to block caspase activation and increase ERK activation^19^. Group B, surprisingly, consists of the previously unrelated compounds diacylglycerol kinase inhibitor II (DKI) and meclizine. Some compounds, such as 4-deoxypyridoxine (DOP) and cysteamine, can be separated from the STHdh^Q111^ vehicle control samples only in the metabolite data, but do not cluster tightly with other compounds. Compounds that clustered together did not have the most similar structures, calculated using the maximum common substructure Tanimoto coefficients in ChemMine tools (Fig. S2)^20^. Likewise, compound pairs with the strongest connectivity scores, as reported by the Connectivity Map using their L1000 gene expression data, did not cluster together in the omics data (Fig. S3)^3^.

To further characterize the compounds in Groups A and B, we performed global proteomic and phosphoproteomic analysis. We identified and measured the levels of 6,281 proteins and 2,560 phosphosites in control and compound-treated cells. We selected two compounds from Group A, cyproheptadine and loxapine, and the two compounds in Group B because they had the most RNA and metabolite changes compared to the STHdh^Q111^ vehicle controls. These four compounds show several statistically significant differentially expressed proteins and phosphosites, and they also cluster reproducibly by their respective groups in both types of proteomic data (Fig. 3B). The differential genes and proteins of the Group A compounds are significantly enriched (FDR-adjusted p-value < 0.05) in 882 and 2 gene ontology (GO) processes, respectively (Tables S2 and S4). The Group B differential genes are significantly enriched (FDR-adjusted p-value < 0.05) in 911 GO processes, but the Group B differential proteins have no significant GO process enrichment (Table S5). Using the IMPaLA tool for metabolite pathway analysis, the Group A and Group B differential metabolites are significantly enriched (FDR-adjusted p-value < 0.05) in 82 and 42 pathways, respectively (Tables S3 and S6).

### Machine learning network models prioritize HD-relevant modes of action

Analyzed separately, the omics data provide a confusing perspective of the changes associated with each compound, pointing to hundreds of potential pathways and processes. To develop a comprehensive view of the compounds’ downstream effects, we turned to dimensionality reduction approaches that leverage known molecular interactions. PIUMet and Omics Integrator use network optimization to identify a subset of the input features that can be linked to each other through direct or indirect molecular interactions^21,22^. We first applied PIUMet to map untargeted metabolomics to the interactome.

To identify the regulatory factors driving changes in transcription, we profiled the H3K4me3 epigenetic modification, which is associated with promoter regions in accessible chromatin, using ChIP-Seq^23^. Though we found few differential peaks between STHdh^Q111^ control cells and compound-treated cells, we used the overall epigenetic signature as a measurement of transcription factor binding accessibility. We predicted transcription factors using a motif analysis approach applied to the differentially expressed genes and the H3K4me3 regions.

**Figure 4:**
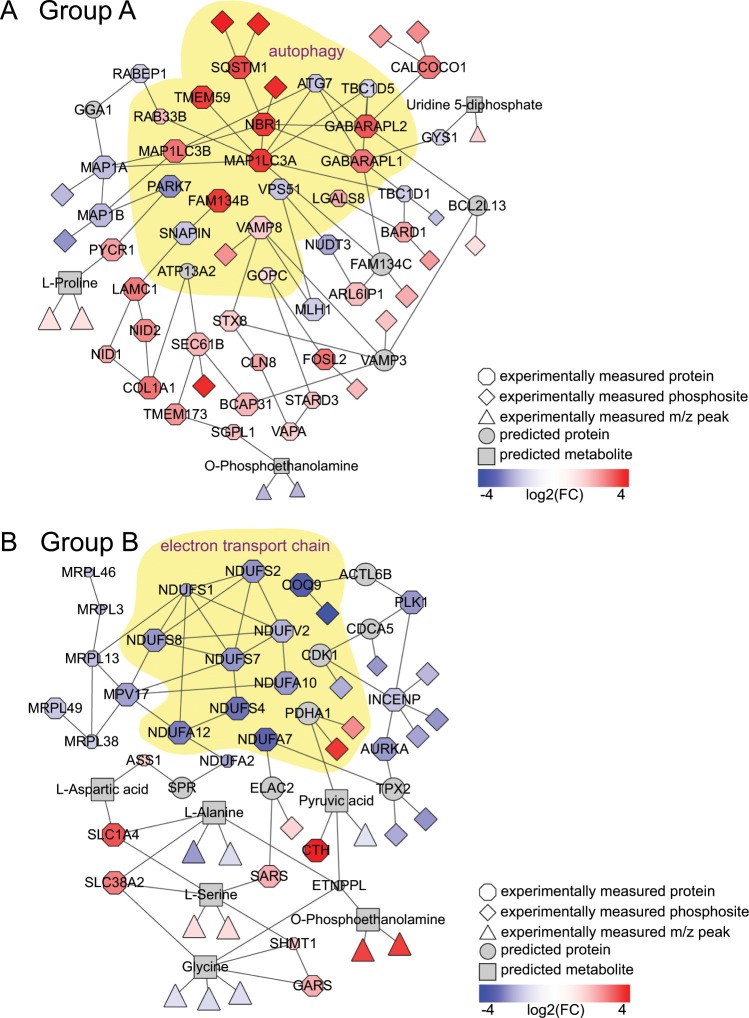
Machine learning network models prioritize HD-relevant pathways. (**A**) The autophagy pathway is significantly enriched (p-value < 0.05) in the Group A compound network, a subnetwork of which is shown. The highlighted yellow region indicates those proteins that are part of the autophagy GO term. (**B**) The electron transport chain is significantly enriched (p-value < 0.05) in the Group B compound network, a subnetwork of which is shown. The highlighted yellow region indicates those proteins that are part of the electron transport chain GO term, part of the mitochondrial respiration pathway. See also Tables S7 and S8.

We then applied Omics Integrator for graph-constrained dimensionality reduction. The inputs were the differential metabolites, proteins, phosphoproteins, and predicted transcriptional regulators for each of the two compound groups. After filtering the networks based on node robustness and specificity, we found significant GO enrichment for pathways relevant in HD. The Group A network was highly enriched for the autophagy, protein localization and transport, and cytoskeleton organization processes (Table S7). The Group B network was highly enriched for the mitochondrial electron transport, sterol metabolism, and amino acid processes (Table S8). Based on the network enrichment, we prioritized the autophagy and mitochondrial respiration pathways for further experimental testing (Fig. 4A,B).

### Autophagy is up-regulated by Group A compounds

**Figure 5:**
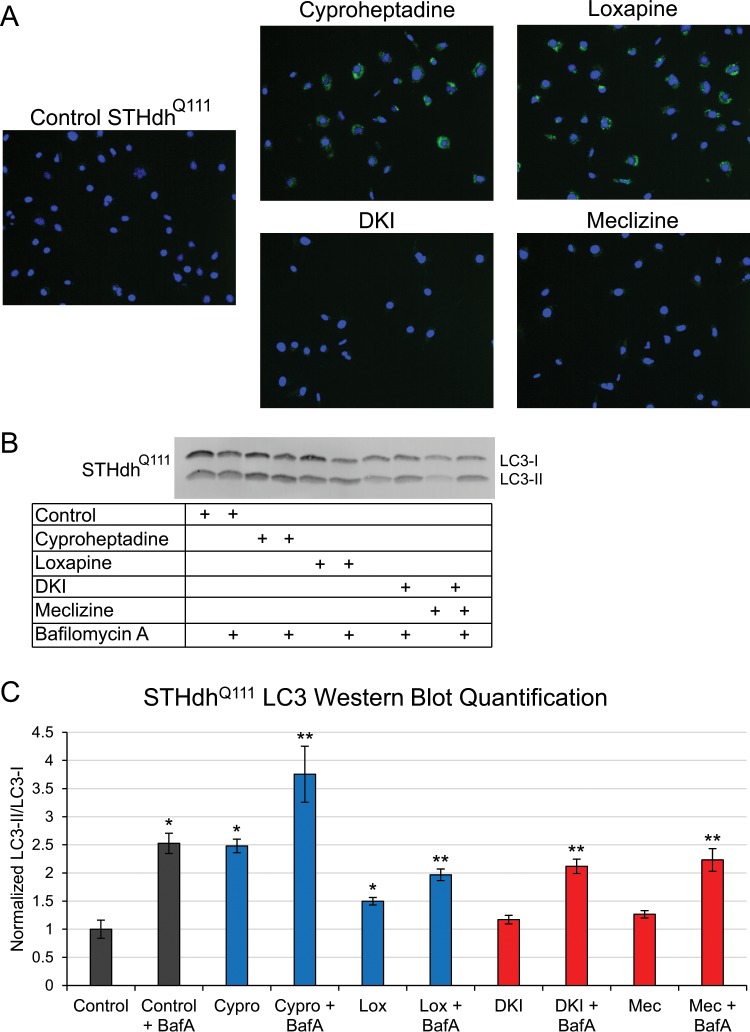
Autophagy is up-regulated by Group A compounds in murine STHdh^Q111^ cells. (**A**) Fluorescent staining of autophagic vacuoles in Group A compound-treated cells compared to Group B compound-treated or control cells. Blue fluorescence indicates nuclei and green fluorescence indicates autophagic vacuoles. (**B**) A representative western blot showing LC3-II and LC3-I levels to determine how the compounds affect autophagy. BafA was used to determine whether the compounds activate autophagy or inhibit vacuole degradation. The full-length western blot is presented in Fig. S5A. (**C**) Quantification of the LC3-II to LC3-I ratio normalized to the control from the western blot. Data are represented as mean ± SD. *p-value < 0.05 compared to Control; **p-value < 0.1 compared to condition-matched non-BafA treatment.

Autophagy, which appeared in the optimized networks, is also known to be dysregulated in HD^24^. We measured the levels of autophagic vacuoles in the STHdh^Q111^ cells using fluorescent staining. We found that the Group A compounds significantly increased the fluorescence intensity, indicating an increase in the number of autophagic vacuoles (Fig. 5A). To further quantify autophagy differences, we examined levels of microtubule-associated protein light chain 3 (LC3), which is widely used to monitor autophagy^25^. We quantified the levels of LC3-II and LC3-I by western blots in control and compound-treated cells. We found a significant increase in the LC3-II to LC3-I ratio with treatment of the Group A compounds, but no significant change with treatment of the Group B compounds (Fig. 5B,C), indicating that the Group A compounds increase formation of autophagic vacuoles. To determine whether this increase was due to an activation of autophagy or a degradation blockage of the autophagic vacuoles, we treated the cells with and without bafilomycin A1 (BafA), an inhibitor of late-stage autophagy^25^. We found a further increase in the LC3-II to LC3-I ratio in all of the conditions upon treatment of BafA, indicating that the Group A compounds activate autophagy in the STHdh^Q111^ cells.

**Figure 6:**
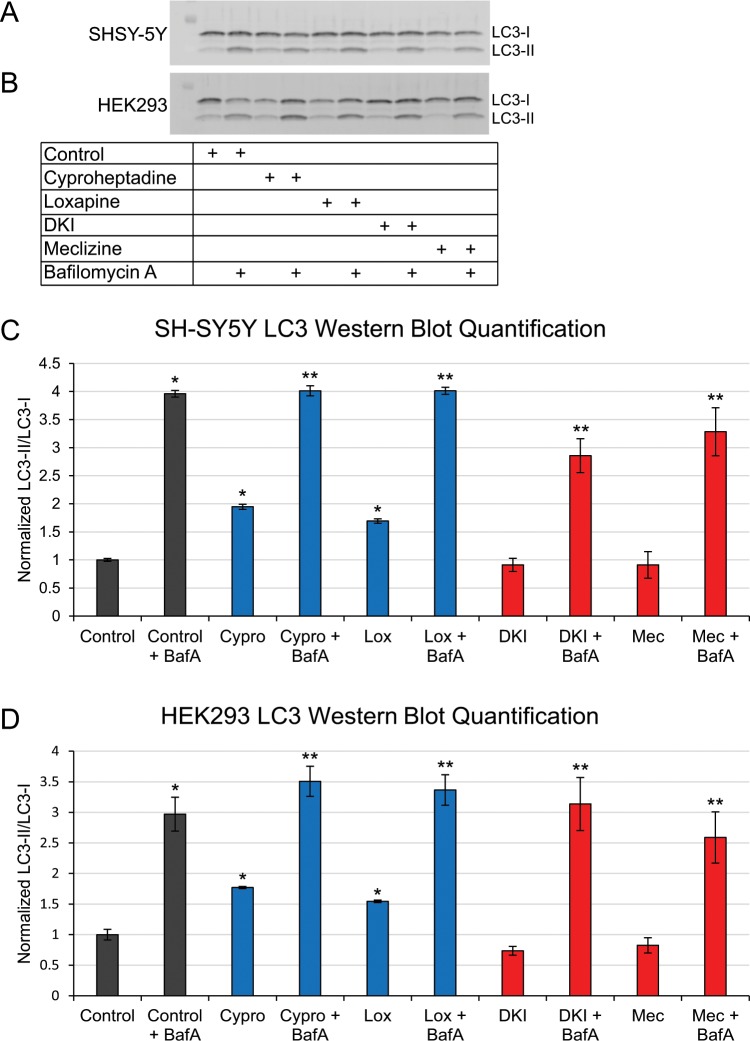
Autophagy is up-regulated by Group A compounds in human SH-SY5Y and HEK293 cells. (**A**) A representative western blot showing LC3-II and LC3-I levels to determine how the compounds affect autophagy in SH-SY5Y cells. The full-length western blot is presented in Fig. S5B. (**B**) A representative western blot showing LC3-II and LC3-I levels to determine how the compounds affect autophagy in HEK293 cells. The full-length western blot is presented in Fig. S5C. (**C**) Quantification of the LC3-II to LC3-I ratio normalized to the control in SH-SY5Y cells from the western blot. *p-value < 0.05 compared to Control; **p-value < 0.1 compared to condition-matched non-BafA treatment. (**D**) Quantification of LC3-II to LC3-I ratio normalized to the control in HEK293 cells from the western blot. Data are represented as mean ± SD. *p-value < 0.05 compared to Control; **p-value < 0.1 compared to condition-matched non-BafA treatment. See also Fig. S4.

As STHdh^Q111^ cells derive from a mouse model of HD, we also tested whether the MoA was relevant in human cells. In human neuronal SH-SY5Y cells, the fluorescent staining assay showed an increase in the number of autophagic vacuoles in the Group A compound-treated cells compared to control cells (Fig. S4). Similar results were obtained in HEK293 cells, which are also human but non-neuronal. In both cell types, the Group A compounds significantly increase the LC3-II to LC3-I ratio, while the Group B compounds do not significantly change the ratio (Fig. 6A–D). The addition of BafA further increased the ratio in all conditions, indicating an activation of autophagy by the Group A compounds in all three cell types.

### Bioenergetics are altered differently by each group of compounds

**Figure 7:**
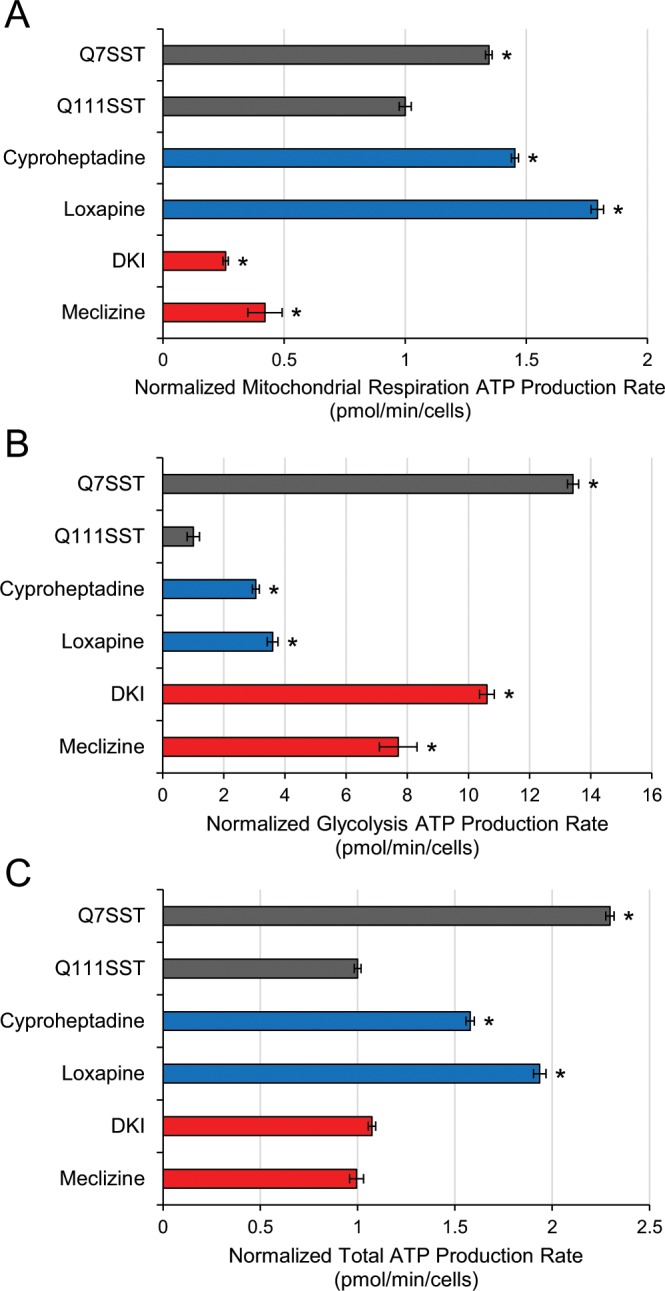
Bioenergetics are altered differently by Group A and Group B compounds in STHdh^Q111^ cells. (**A**) Quantification of the mitochondrial respiration ATP production rate normalized to the STHdh^Q111^ control. (**B**) Quantification of the glycolysis ATP production rate normalized to the STHdh^Q111^ control. (**C**) Quantification of the total ATP production rate normalized to the STHdh^Q111^ control. Data are represented as mean ± SEM. *p-value < 0.05.

The network analysis of Group B compounds suggested an MoA relating to bioenergetics, which are also known to be affected in HD^26^. To test this, we used the Seahorse Real-Time ATP Production assay to measure the rates of mitochondrial respiration and glycolysis in STHdh^Q111^ control and compound-treated cells. We found that both Group B compounds indeed inhibited mitochondrial respiration and enhanced glycolysis compared to the control cells, but the total ATP production levels were unchanged (Fig. 7A–C). Interestingly, we also found significantly enhanced mitochondrial respiration and slightly enhanced glycolysis ATP production rates by the Group A compounds. The net ATP production was increased by the Group A compounds compared to the STHdh^Q111^ control cells. The two groups of compounds show seemingly opposite effects, where the Group A compounds primarily rescue the mitochondrial respiration deficit and the Group B compounds rescue the glycolysis deficit present in the STHdh^Q111^ cells compared to the STHdh^Q7^ cells.

## Discussion

The molecular effects of drug candidates are complex and can be difficult to interpret. Cataloguing efforts, such as those by the Connectivity Map, LINCS and Genomics of Drug Sensitivity in Cancer consortia, have made it possible to rapidly compare small molecules using expression or bioactivity data^1–3,9,27–29^. In cases where a compound of interest shows similarities to one with known MoAs, this process can lead to functional insights. However, these compendia themselves contain thousands of compounds that do not match up to any reference.

Our findings demonstrate the value of an approach that combines multi-omics with an interpretable machine learning method to determine previously unknown MoAs, even in the absence of a comparable reference. We identified and experimentally validated Huntington’s Disease-relevant MoAs for two classes of compounds. Although the 30 compounds tested in this study were previously shown to reverse an HD phenotype, their disease-relevant MoAs were unknown. Analyzing the 14 protective compounds, we found unexpected similarities in their molecular effects. These clusters of compounds would not have been predicted solely based on the compounds’ phenotypic viability readouts, structural similarities, connectivity scores, or known binding targets (Figs. 2A, S2, S3).

Using the network results to guide experiments, we confirmed that autophagy is activated by Group A compounds and that mitochondrial respiration is inhibited by Group B compounds in the STHdh^Q111^ cells (Figs. 5A–C and 7A–C). The specific effects on autophagy, mitochondrial respiration, and glycolysis by the Group A compounds were previously unknown. We verified that the Group A compounds activate autophagy in other cell lines, namely human SH-SY5Y and HEK293 cells (Figs. 6A–D, S2A,B). We note that in the autophagy results, the LC3-II/LC3-I ratio with BafA alone is higher than that of the combination of loxapine and BafA (Fig. 5B,C). This observation suggests that there are interactions between the two compounds. There are many possible explanations for the nature of this interaction that will require further investigation. We speculate that loxapine could be making BafA less effective by overwhelming the block of autophagosome degradation through the activation of autophagy. It has been reported that the Group B compound meclizine is an inhibitor of mitochondrial respiration and activator of glycolysis, which we confirmed^30,31^. The other Group B compound, DKI, has not previously been associated with changes in bioenergetics, but has the same effect as meclizine in the STHdh^Q111^ cells. Though our multi-omics machine learning approach can identify a compound’s MoAs, it does not pinpoint the precise changes in the pathways required to produce the compound’s effect. Future experimental efforts to modulate specific parts of the autophagy and bioenergetic pathways could lead to an increased understanding of the compounds’ effects. The effects on these pathways could even be related as these pathways are not distinct biological processes. For example, mitochondrial respiration will be affected by mitophagy, a form of autophagy. The omics data we collected in this study are available and can be used to guide drug repurposing efforts (Materials and Methods). For example, we hypothesize that the Group A compounds could be strong drug candidates for diseases where autophagy is deficient, like in neurodegenerative diseases.

It is important to note that the disease-relevant MoA might be distinct from that previously reported in the literature. Cyproheptadine and meclizine are both antihistamines known to antagonize the histamine H1 receptor^29^. Based on their reported MoAs and their effectiveness in HD models, these two antihistamines might have been expected to have similar therapeutic mechanisms. However, the two compounds have dissimilar omics profiles and fall in different clusters (Fig. 3A). Indeed, our machine learning approach predicted that they would have different effects on autophagy and bioenergetics, which we confirmed experimentally. On the other hand, our approach suggested, and we experimentally confirmed, a common disease-relevant MoA for DKI and meclizine, whose reported targets (diacylglycerol kinase and histamine H1 receptor, respectively) are unrelated. Thus, the phenotypic effects of a compound can be unpredictable even when a direct target is known.

Our work also highlights the importance of using complementary omics assays. Groups A and B affected a similar number of genes and are nearly indistinguishable in their RNA profiles (Fig. 2A). For these compounds, metabolite profiling data proved more useful than the gene expression data in revealing their different effects. These distinct groups are reproduced in the proteomic data and ultimately reflect the functional differences in biological processes, such as in the autophagy and bioenergetics pathways. Metabolomic assays are less expensive than proteomic assays, but unlike the transcriptomic data, they still provide the resolution needed to suggest differences between the two groups of compounds. However, other compounds showed little to no effect on metabolites, but did robustly alter gene expression. It is also noteworthy that though the clusters of compounds were the same, the Group A compounds affected more proteins and phosphosites than the Group B compounds, whereas the Group B compounds affected more lipids and polar metabolites than the Group A compounds (Fig. S1). Thus, there may be no single omic method that will provide sufficient data for all compounds.

An interpretable machine learning approach was essential for identifying the cellular processes underlying the omics effects. The physical interaction networks allowed us to identify and prioritize the autophagy and mitochondrial respiration pathways as processes affected by the Group A and Group B compounds, respectively (Fig. 4A,B). These pathways were not top hits in the gene, metabolite, or protein enrichments for either group of compounds (Tables S2–S6). In each data type alone, there are hundreds to thousands of changes and no direct mechanistic insight, but physical interaction models enable identification of compounds’ MoAs.

The multi-omics machine learning method we describe can be broadly applied to any disease and virtually any type of data. Because the machine learning leverages a molecular interaction network containing metabolites, proteins, transcriptional regulators and genes, it was equally effective in revealing MoAs for drugs that target metabolic processes and those that affect autophagy. Inference based on individual metabolites would need further validation of metabolite identities, but our method draws an inference from integration of the putative metabolite matches and the other omics data. As in PIUMet, we rely on the inferred pathway instead of any specific assignment of an individual metabolite^21^. Our approach compensates for the inherent uncertainty in the data by inferring pathways from the integration of many data points. The network approach can easily be extended to incorporate other molecular data, including genetics, as we have shown previously^22,32–36^.

Methods that seek to match an unknown compound with those in a library of molecular signatures are always limited by the scope of the training data. In many cases, these data are heavily biased toward studies of experimentally convenient systems, often focusing on gene expression and cancer cell lines. By contrast, our approach focuses on data from the compounds of interest in their disease context and can thus be applied to less commonly studied diseases and cell types. Wider adoption of multi-omics data collection and interpretable predictive models hold great potential for accelerating drug development.

## Methods

### STHdh cell lines

Conditionally immortalized wild-type STHdh^Q7^ (female, Coriell CH00097, RRID: CVCL_M590) and mutant huntingtin homozygous knock-in STHdh^Q111^ (female, Coriell CH00095, RRID: CVCL_M591) murine striatal progenitor cell lines were purchased from Coriell. Cells were maintained at 33 °C with 5% CO_2_ and cultured in Dulbecco’s modified Eagle’s medium (DMEM, Corning 10-013) supplemented with 10% fetal bovine serum (FBS, Gemini Bio-Products 100–106), and 1% penicillin/streptomycin (Gemini Bio-Products 400–109).

### SH-SY5Y cell line

Human neuroblastoma SH-SY5Y (ATCC® CRL-2266™, female, RRID: CVCL_0019) cells were purchased from ATCC. Cells were maintained at 37 °C with 5% CO_2_ and cultured in a 1:1 mixture of ATCC-formulated Eagle’s Minimum Essential Medium (ATCC 30–2003) and F12 medium (ThermoFisher Scientific 11765-054) supplemented with 10% fetal bovine serum (Gemini Bio-Products 100–106).

### HEK293T/17 cell line

Human embryonic kidney HEK293T/17 (ATCC® CRL-11268™, female, RRID: CVCL_1926, referred to as HEK293 in text) cells were purchased from ATCC. Cells were maintained at 37 °C with 5% CO_2_ and cultured in Dulbecco’s modified Eagle’s medium (DMEM, Corning 10–013) supplemented with 10% fetal bovine serum (FBS, Gemini Bio-Products 100–106), 1% penicillin/streptomycin (Gemini Bio-Products 400–109), and L-glutamine (Sigma-Aldrich G7513).

### Compound treatment

STHdh cells were incubated in serum-free medium with a compound or vehicle control (DMSO, Sigma-Aldrich 67-68-5) for 24 hours. We chose a treatment time of 24 hours because of the time required to produce a significant cell death phenotype in the STHdh^Q111^ cells. SH-SY5Y and HEK293 cells were incubated in their respective complete medium with a compound or vehicle control (DMSO, Sigma-Aldrich 67-68-5) for 24 hours. The compounds were dissolved in DMSO or water before being added to each medium. For some of the autophagy western blot samples, we also treated the cells for 2 hours with 100 nM bafilomycin A1 (Sigma-Aldrich B1793).

### Viability assay

Cell viability was measured using high-content imaging. STHdh^Q111^ cells were seeded at 6,000 cells/well in black 96-well microplates. After 24 hours, the cells were treated with a compound or vehicle. After another 24 hours, 1 ug/mL calcein-AM (ThermoFisher Scientific C3099), 2 ug/mL propidium iodide (PI, ThermoFisher Scientific P3566), and 1.5 ug/mL Hoechst 33442 (ThermoFisher Scientific H3570) were added to detect and quantify live, dead, and total cells, respectively. After a 20-minute incubation, the Cellomics Arrayscan Platform (ThermoFisher Scientific) was used for image acquisition and quantitative analysis. ImageJ was used to create composite images^37^. STHdh^Q7^ cells with vehicle were also tested using the same procedure, but with a seeding density of 4,500 cells/well to account for the differences in growth rate between the cell lines. From the fluorescent images of labeled cells, cell death was quantified as the ratio of PI-positive cells to Hoechst-positive cells using CellProfiler^38^. Three independent 96-well plates with ten replicate wells each were conducted for each compound and multiple concentrations spanning at least three orders of magnitude were tested. The concentration at which there is minimal cell death is reported for each compound (Table S1). For each experiment, a Student’s t-test was applied, and Fisher’s method was used to combine the independent experiments and determine significance with a p-value threshold of 0.001. A protective compound in the STHdh^Q111^ model is defined as one that significantly decreased the amount of cell death compared to STHdh^Q111^ vehicle control.

### RNA-Seq

RNA was extracted from compound- or vehicle-treated cells in triplicate using Zymo Research Quick-RNA™ MiniPrep (Plus) kit (Zymo Research R1058) and RIN values were tested using Advanced Analytical. All samples had RIN values greater than 0.85. Libraries were prepared using NEBNext® Ultra™ Directional RNA Library Prep Kit for Illumina® (New England Biolabs E7420L) and NEBNext® Poly(A) mRNA Magnetic Isolation Module kit (New England Biolabs E7490L). Libraries were multiplexed and sequenced on an Illumina Hi-Seq. 2000 for single-end 50 bp reads. Adapter sequences were trimmed from sequencing reads using Trimmomatic-0.36^39^. Reads were aligned to the GRCm38.p5 transcriptome (https://www.gencodegenes.org/mouse/release_M12.html) and quantified using RSEM^40^. DESeq. 2 with batch effect modeling by collection day was used to find differentially expressed genes for each compound treatment compared to STHdh^Q111^ vehicle control^41^. The differentially expressed genes were filtered using a Benjamini-Hochberg corrected p-value threshold of 0.05.

### Untargeted metabolomics

STHdh^Q111^ cells were grown on 10 cm dishes in triplicate at a seeding density of 1.06 million cells/well. Compound- or vehicle-treated cells were washed with cold 0.9% NaCl. To each 10 cm dish of cells, 660 uL LC/MS-grade methanol containing internal standards and 330 uL LC/MS-grade water were added. Cells were scraped and transferred to Eppendorf tubes, where 450 uL chloroform was added. Samples were vortexed at maximum speed (20,817 rcf) for 10 minutes at 4 °C. Each layer was collected separately, avoiding the precipitate at the interface of the two layers, and dried by speedvac. Lipid and polar metabolite profiling were performed by members of the Whitehead Institute Metabolite Profiling Core Facility. Metabolite quantification in positive and negative ionization mode was log2 normalized and analyzed using limma with batch effect modeling by collection day, and differentially expressed metabolites were filtered using a Benjamini-Hochberg corrected p-value threshold of 0.05^42^. Untargeted metabolite m/z peaks were matched to known metabolites using PIUMet, with a metabolite database compiled using HMDBv4.0 and Recon3D^21,43,44^.

### H3K4me3 ChIP-seq

Compound- or vehicle-treated cells were crosslinked with 1% formaldehyde for 8 minutes and quenched with glycine for 5 minutes, lysed in 2X lysis buffer (50 mM Tris-HCl pH8, 150 mM NaCl, 1% Triton X-100, 0.1% Na Deoxycholate, 5 mM CaCl2 and protease inhibitors) for 20 minutes on ice, and digested with 100 u MNase (New England Biolabs M0247) for 10 minutes at 37 °C. The MNase digestion was terminated by addition of 10 mM EDTA. Chromatin was incubated with the anti-H3K4me3 antibody (Millipore 07–473, RRID: AB_1977252) overnight at 4 °C, followed by incubation with Protein G beads (Invitrogen 10004D) for 2 hours at 4 °C. The beads were washed with PBS (6×) and samples were eluted in EB (10 mM Tris-HCl pH8, 5 mM EDTA, 300 mM NaCl, 0.1% SDS) supplemented with Proteinase K (New England Biolabs P8107S). SPRI beads were used for clean-up and yield was measured using Qubit Fluorimeter. Libraries were prepared using NEBNExt® Ultra™ II DNA Library Prep Kit for Illumina (New England Biolabs E7645S). Libraries were sequenced on an Illumina Hi-Seq. 2000 for single-end 50 bp reads.

### Proteomics

Proteomics were performed by members of the Thermo Fisher Scientific Center for Multiplexed Proteomics at Harvard Medical School. Proteomic data was collected from cells treated with Group A compounds, Group B compounds, or vehicle controls in triplicate. Please see Weekes *et al*., McAlister *et al*., and below for detailed descriptions of the assay^45,46^. In brief, sample processing steps included cell lysis, tandem protein digestion using LysC and trypsin, peptide labeling with Tandem Mass Tag 6-plex reagents, IMAC enrichment of phosphopeptides, and peptide fractionation. Multiplexed quantitative mass spectrometry data were collected on an Orbitrap Fusion or Lumos mass spectrometer operating in an MS3 mode using synchronous precursor selection for the MS2 to MS3 fragmentation. Using the SEQUEST algorithm, MS/MS data were searched against a Uniprot mouse database with both the forward and reverse sequences. Additional data processing steps included controlling peptide and protein level false discovery rates, assembling proteins from peptides, and protein quantification from peptides. Phosphosite quantification was normalized to protein quantification, and both protein and phosphosite data were then log2 normalized and analyzed using limma^42^. Differentially expressed proteins and phosphosites were filtered using a Benjamini-Hochberg corrected p-value threshold of 0.05.

### Network analysis

Differential proteins, phosphosites, m/z lipid and polar metabolite peaks, and predicted transcription factors for each compound treatment compared to vehicle control were mapped onto the interactome, comprised of physical interactions between proteins (iRefIndex v14), proteins and metabolites (HMDBv4.0, Recon3D), phosphosites and kinases (PhosphositePlus), m/z peaks and matched metabolites (PIUMet), and phosphosites and proteins^21,43,44,47,48^. The Prize-Collecting Steiner Forest (PCSF) algorithm was applied using Omics Integrator 2 to find the set of highly relevant pathways associated with each compound treatment^22^. PCSF was run 100 times with random noise on the edges for robustness measurements and random input sets for specificity measurements. The optimal network solution was filtered by those nodes with at least 40% robustness and specificity.

### Autophagic vacuole fluorescence staining

Compound-treated and untreated STHdh^Q111^ and STHdh^Q7^ cells were seeded at 6,000 cells/well in black 96-well microplates. After 24 hours, compounds or vehicle controls were added. After a further 24 hours, the Autophagy Detection Kit (Abcam ab139484) was used to measure autophagic vacuoles in living cells, according to the manufacturer’s instructions. Hoechst 33442 (ThermoFisher Scientific H3570) was used to stain the nuclei of cells. Cells with activated autophagy had bright green fluorescent signal. ImageJ was used to create composite images^37^. Assay conditions for the SH-SY5Y and HEK293 cells were similar, but with initial seeding concentrations of 25,000 and 5,000 cells/well, respectively.

### Western blots

To quantify LC3 protein expression, adherent cells were scraped in 200 μl ice-cold RIPA buffer (50 mM Tris‐HCl pH 8.0, 150 mM NaCl, 1% Triton X-100, 0.5% Sodium Deoxycholate, 0.1% SDS supplemented with freshly made protease inhibitors (cOmplete™, EDTA-free Protease Inhibitor Cocktail, Sigma-Aldrich 11873580001)). Samples were incubated with agitation for 30 min at 4 °C and centrifuged at 12,000 × g for 20 min at 4 °C. The supernatant, containing the protein extracts, was collected. Protein concentration was measured with the Bradford Assay. Protein lysates were separated using SDS/PAGE electrophoresis and transferred to a PVDF membrane. The membranes were rinsed and blocked for 1 hour at room temperature and incubated overnight with primary antibodies in blocking solution with 0.1% Tween-20. The following primary antibodies were used: anti-LC3B (Sigma-Aldrich L7543, dilution 1:500, RRID: AB_796155); anti-actin (Abcam 1801, dilution 1:1000). The membranes were washed and incubated at room temperature for 1 hour with a secondary antibody in a 1:1 PBS, blocking buffer solution with 0.1% Tween-20. The following secondary antibody was used: 800CW Donkey anti-Rabbit IgG (Li-Cor Biosciences 925–32213, dilution 1:10000, RRID: AB_2715510). The membranes were rinsed and scanned using the Odyssey infrared imaging system (Li-Cor Biosciences). Protein expression was measured using integrated intensity readings in regions around protein bands.

The LC3-II/LC3-I ratio was calculated for each sample, with and without the addition of bafilomycin A1 (BafA). For each compound condition without BafA, a Student’s t-test was applied to the three replicates of the compound-treated samples compared to the vehicle control samples. Significance was determined with a p-value threshold of 0.05. For each compound condition with BafA, a Student’s t-test was applied to the three replicates of the BafA-treated samples compared to their respective condition’s BafA-untreated samples. Significance was determined with a p-value threshold of 0.1. The LC3-II/LC3-I ratios for samples in each cell line are normalized to their respective controls in the quantification, such that the control samples have a ratio of 1 (Figs. 5 and 6).

### ATP production rate assay

Compound-treated and untreated STHdh^Q111^ and STHdh^Q7^ cells were seeded at 6,000 cells/well in black 96-well microplates. After 24 hours, compounds or vehicle controls were added. After a further 24 hours, the Agilent Seahorse XF Real-Time ATP Rate Assay Kit (Agilent 103592–100) was used to simultaneously measure the rate of ATP production from mitochondrial respiration and glycolysis, according to manufacturer’s instructions. Briefly, assay medium was prepared by supplementing 100 mL of Seahorse XF DMEM Medium, pH 7.4 with 10 mM of XF glucose, 1 mM of XF pyruvate, and 2 mM of XF glutamine. On the day of the assay, the plated cell medium was replaced by assay medium. The sensor cartridge was hydrated the day prior to the assay and kept overnight at 37 °C in a non-CO2 incubator. On the day of the assay, the sensor cartridge was loaded with a final well concentration of 1.5 uM oligomycin and 0.5 uM rotenone/antimycin A. Extracellular acidification rates (ECAR) and real-time oxygen consumption rates (OCR) were measured with the Agilent Seahorse XFe96 analyzer at 33 °C. The oligomycin was injected to inhibit mitochondrial ATP synthesis, which resulted in a decrease in OCR, allowing the mitochondrial respiration ATP rate to be calculated. ECAR data was used to calculate the total proton efflux rate. The injection of rotenone/antimycin A completely inhibited mitochondrial respiration, which allowed for an estimation of mitochondrial-associated acidification. Combined with the proton efflux rate, these measurements were used to calculate the glycolysis ATP production rate. The total ATP production rate was calculated from the sum of the rates of ATP production from mitochondrial respiration and glycolysis. Assay conditions for the SH-SY5Y and HEK293 cells were similar, but with a temperature of 37 °C and initial seeding concentrations of 25,000 and 5,000 cells/well, respectively. For each compound condition, a Student’s t-test was applied to the data for the replicates (at least 6 per treatment) of the compound-treated samples compared to the vehicle control samples.

## Supplementary information

Supplemental Information.

Table S2.

Table S3.

Table S5.

Table S6.

Table S7.

Table S8.

## Data Availability

The datasets produced in this study are available in the following databases: • RNA-Seq data: Gene Expression Omnibus GSE129144 • ChIP-Seq data: Gene Expression Omnibus GSE129144 • Mass spectrometry untargeted metabolomic data: Metabolomics Workbench ST001186 • Mass spectrometry proteomic data: MassIVE MSV000084607
