# Supplementary material for: A Multi-Omics Interpretable Machine Learning Model Reveals Modes of Action of Small Molecules
Source: Sci Rep. 2020 Jan 22;10:954. doi: 10.1038/s41598-020-57691-7 (PMC6976599; doi:10.1038/s41598-020-57691-7)
Supplement: Supplementary file 1 — Supplemental Information. [file 41598_2020_57691_MOESM1_ESM.pdf]

A Multi-Omics Interpretable Machine Learning Model Reveals Modes of Action of Small Molecules

Natasha L. Patel-Murray[1], Miriam Adam[2], Nhan Huynh[2], Brook T. Wassie[2], Pamela Milani[2], and Ernest Fraenkel[2,3,4,*]

[1]Computational and Systems Biology Graduate Program, Massachusetts Institute of Technology, 77 Massachusetts Avenue, Cambridge, MA 02139, USA

[2]Department of Biological Engineering, Massachusetts Institute of Technology, 77 Massachusetts Avenue, Cambridge, MA 02139, USA

[3]Broad Institute, Cambridge, MA 02139, USA

[4]Lead Contact

*Correspondence: fraenkel-admin@mit.edu

<u>Supplemental Information</u>

Supplemental Information includes five figures, eight tables, and detailed methods.

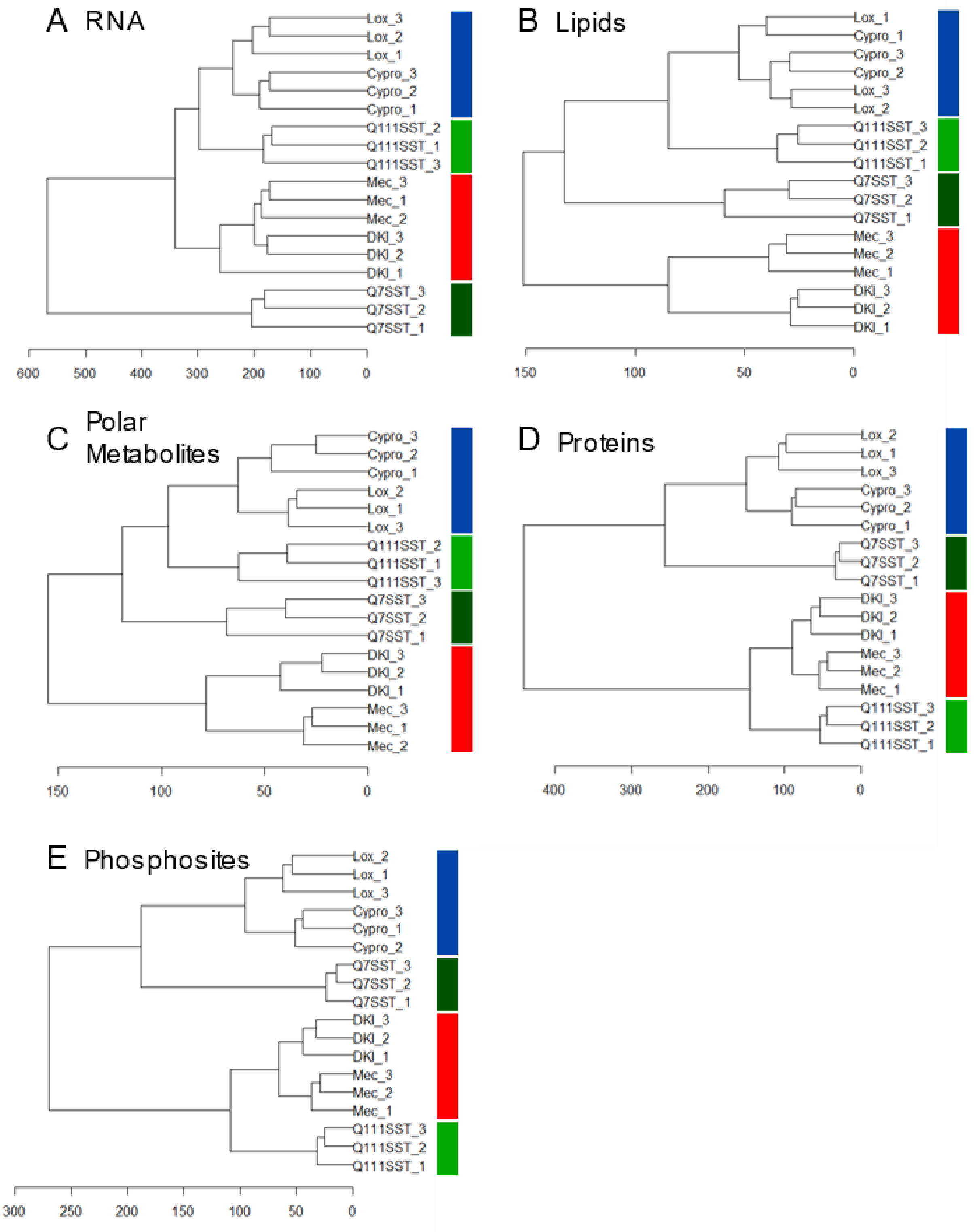

Figure S1. Distinct omics data lead to different clustering patterns between the compound-treated and control samples.

Related to Figure 3.

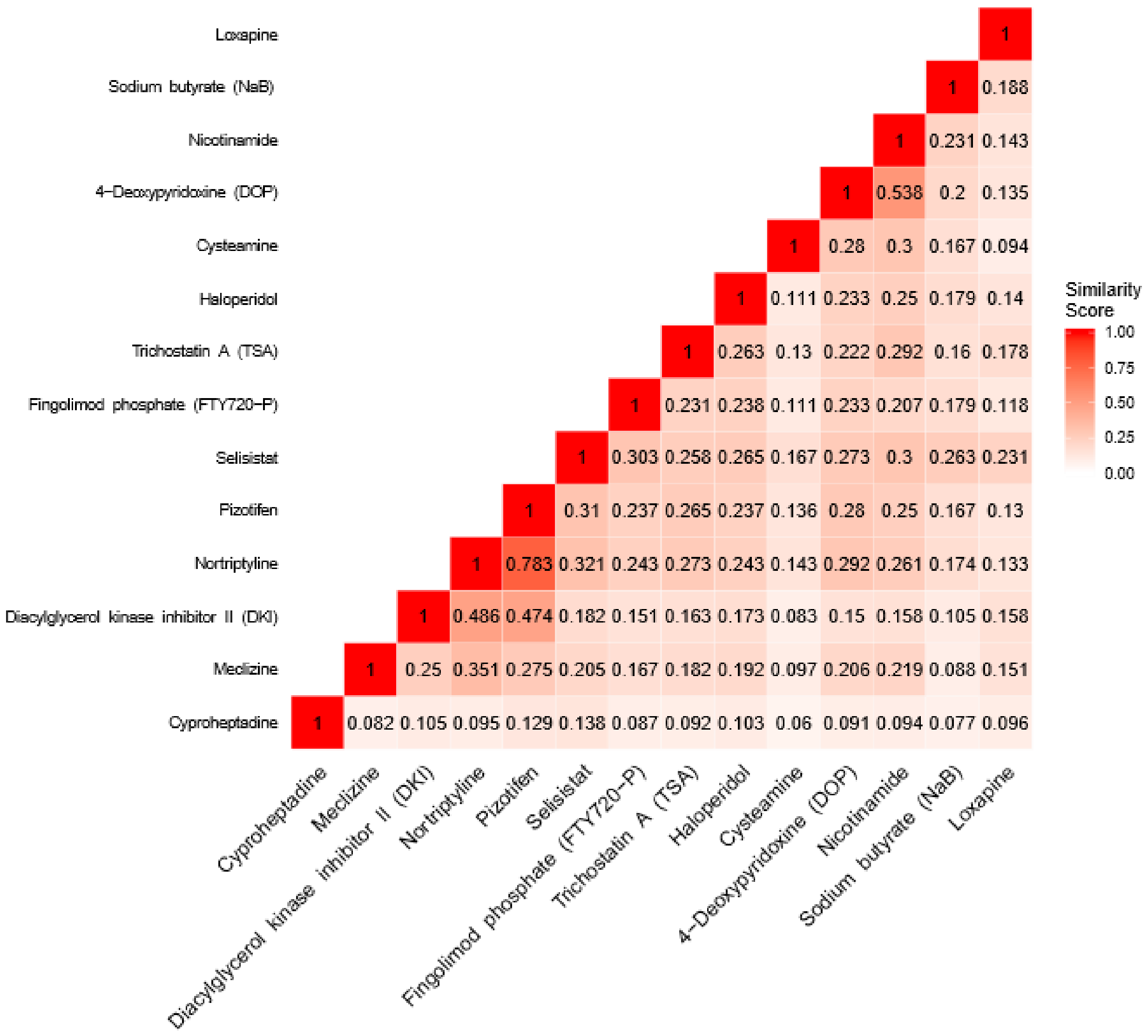

Figure S2. Groupings of compounds would not be predicted based on structural similarities determined by maximum common substructure (MCS) Tanimoto coefficients. Each number within the matrix indicates the similarity score calculated using the MCS Tanimoto coefficients. Cells on the diagonal were assigned a similarity score of 1. Related to Figure 3.

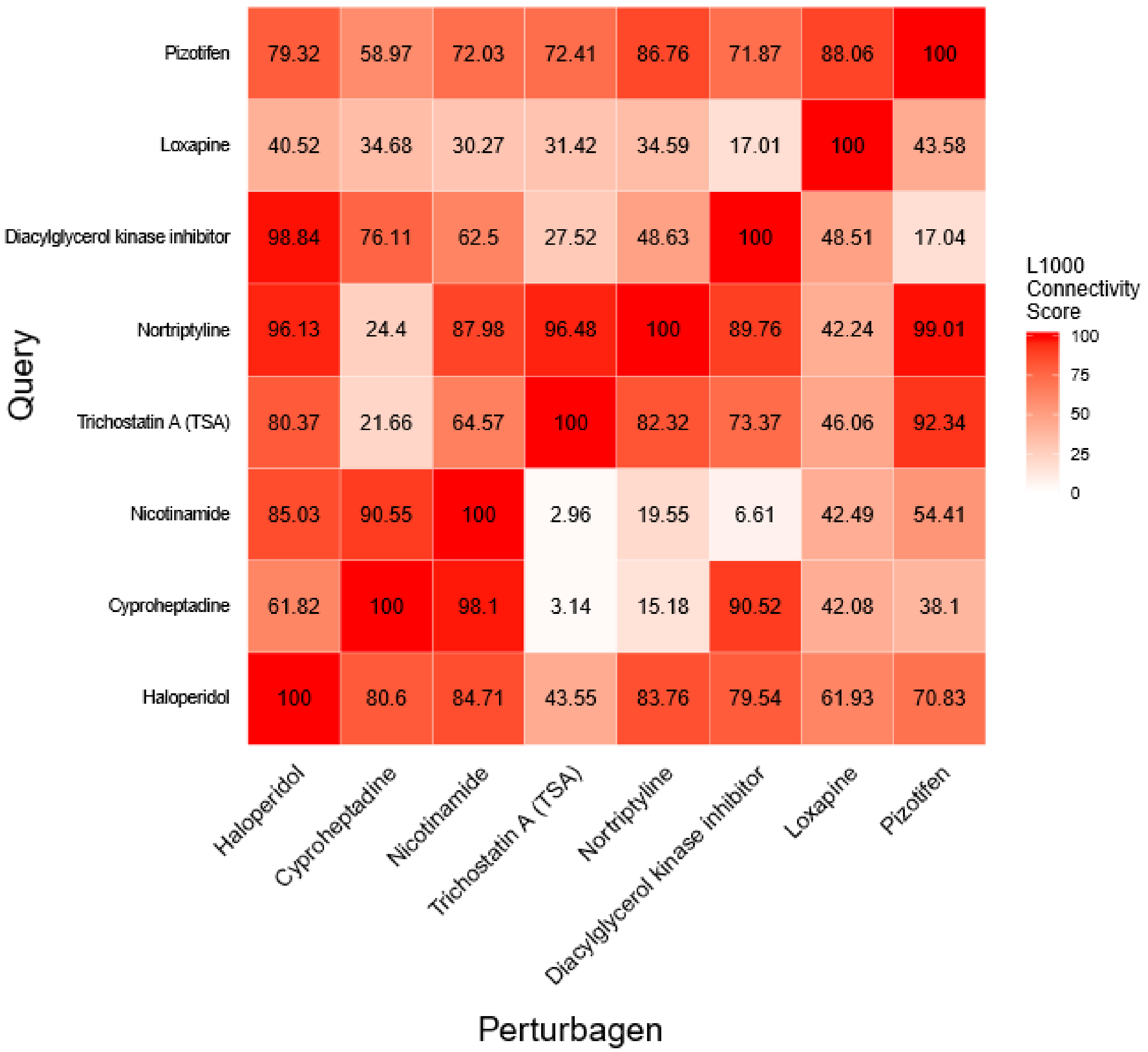

Figure S3. Groupings of compounds would not be predicted based on their L1000 connectivity scores.

Each number within the matrix indicates the connectivity score, where the compound on the y-axis was used as the query and the compound on the x-axis was used as the perturbagen. Cells on the diagonal were assigned a connectivity score of 100. Related to Figure 3.

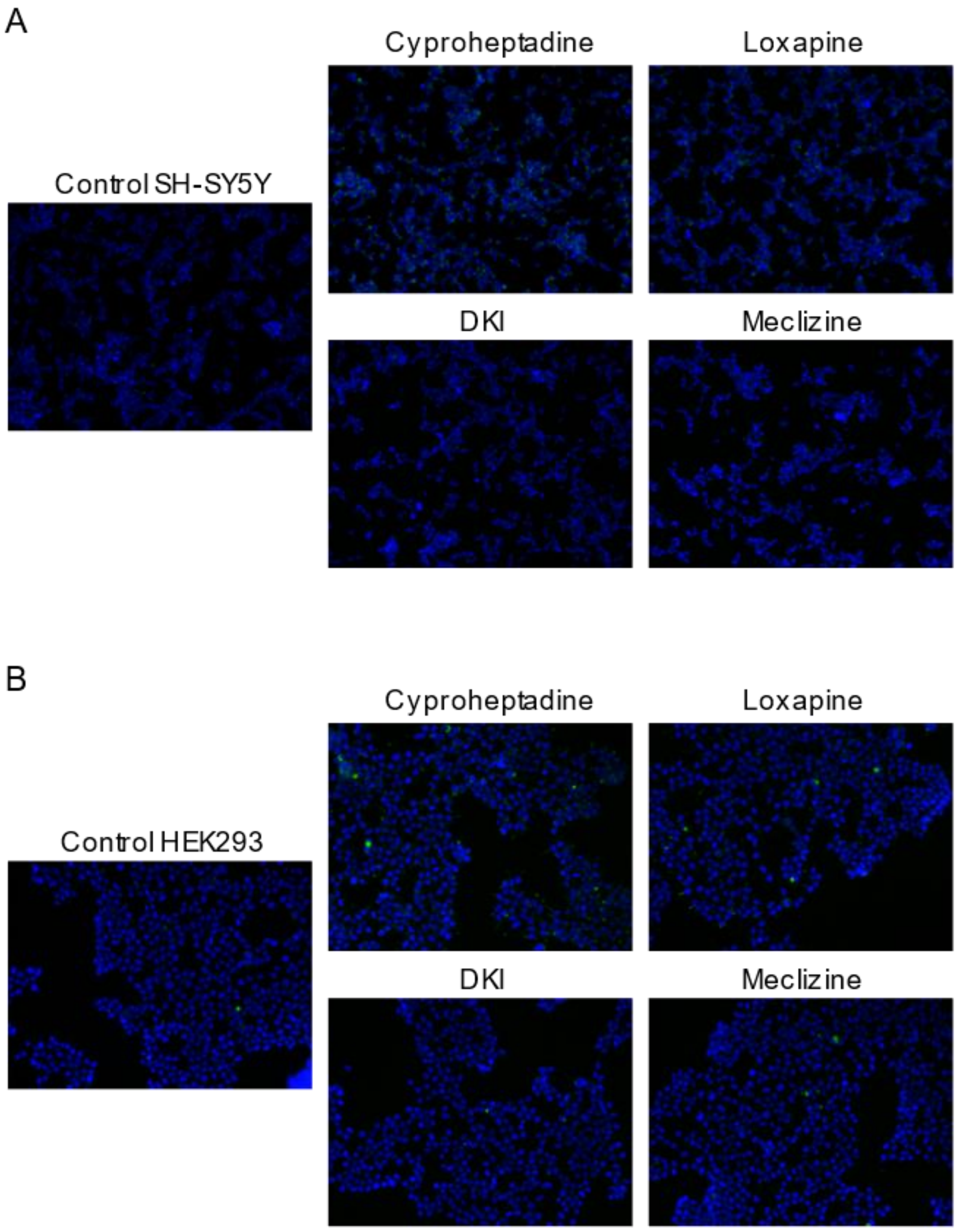

Figure S4. Staining of autophagic vacuoles is increased by Group A compounds in (A) SH-SY5Y and (B) HEK293 cells.
Related to Figure 6.

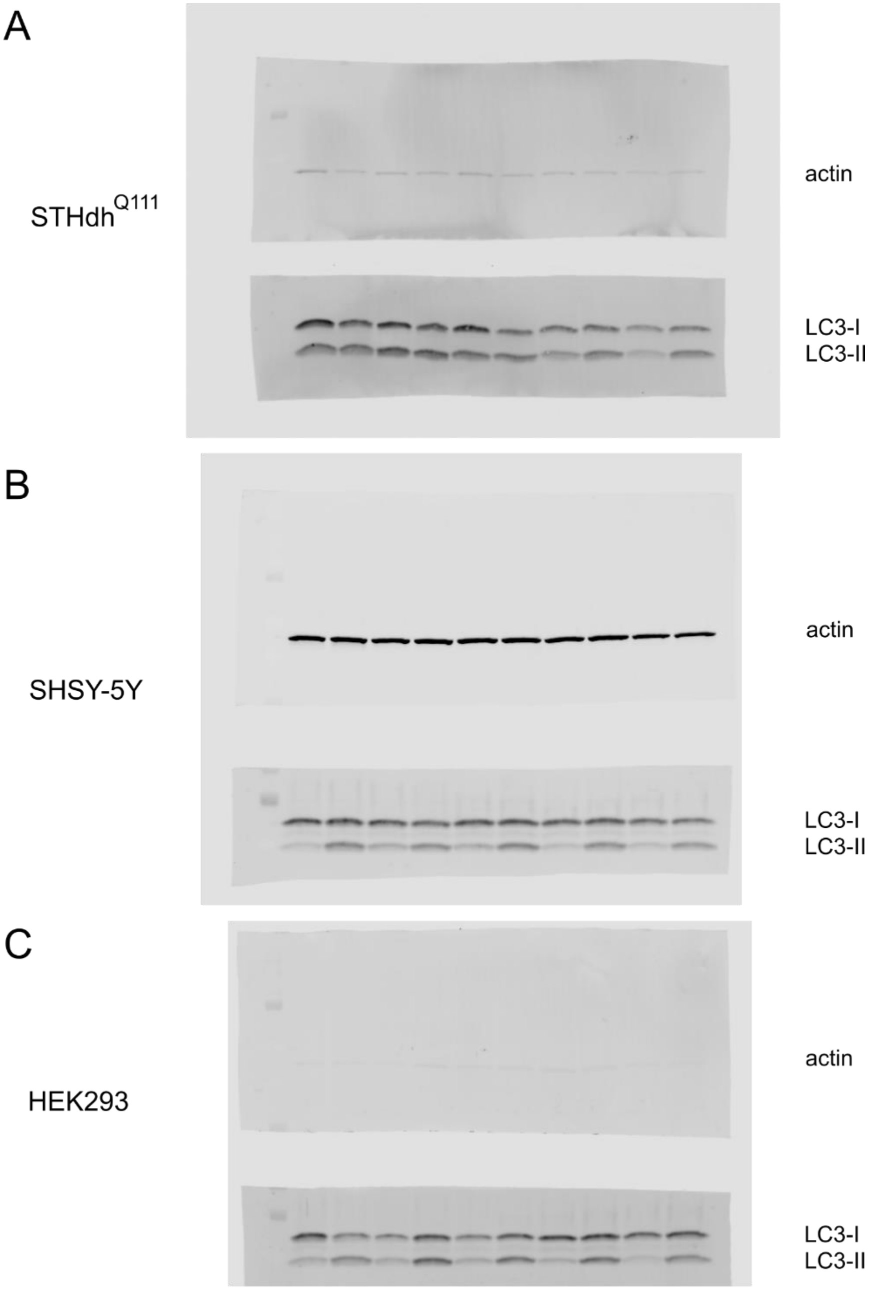

Control	+	+								
Cyproheptadine			+	+						
Loxapine					+	+				
DKI							+	+		
Meclizine									+	+
Bafilomycin A		+		+		+		+		+

Figure S5. Full-length western blot images for (A) $STHdh^{Q111}$, (B) SH-SY5Y, and (C) HEK293 cells. Each blot was cut in two prior to imaging, and both pieces were imaged together.

Related to Figures 5 and 6.

Table S1. Dose, vendor, literature reference, FDA-approval status, and known targets for the 30 tested compounds.
Related to Figure 2.

Compound	Dose	Vendor	Literature Reference	FDA Approval	Known DrugBank Targets
Meclizine	10uM	Sigma-Aldrich	Gohil et al., 2013	Yes	HRH1, NR1I3
Sodium butyrate	1mM	Sigma-Aldrich	Bates, Tabrizi, and Jones, 2014	No	--
Cyproheptadine	10uM	Sigma-Aldrich	Sarantos, Papanikolaou, Ellerby, and Hughes, 2012	Yes	HRH1, HTR2A, HTR2C, CHRM1, CHRM2, CHRM3, HTR7
Loxapine	10uM	Sigma-Aldrich	Sarantos, Papanikolaou, Ellerby, and Hughes, 2012	Yes	DRD2, DRD1, HTR2A, HTR2C, HTR1A, HTR1B, HTR1D, HTR1E, HTR3A, HTR5A, HTR6, HTR7, ADRA1A, ADRA1B, ADRA2A, ADRA2B, ADRA2C, ADRB1, CHRM1, CHRM2, CHRM3, CHRM4, CHRM5, DRD3, DRD4, DRD5, HRH1, HRH2, HRH4, SLC6A4, SLC6A2, SLC6A3
4-Deoxypyridoxine	4mM	Sigma-Aldrich	Pirhaji et al., 2017	No	--
Selisistat	10uM	Selleckchem	Westerberg et al., 2015	No	SIRT1
Trichostatin A	10nM	Sigma-Aldrich	Bates, Tabrizi, and Jones, 2014	No	--
Diacylglycerol kinase inhibitor II	10uM	Sigma-Aldrich	Zhang et al., 2012	No	--
Nicotinamide	0.5nM	Sigma-Aldrich	Bates, Tabrizi, and Jones, 2014	Yes	ETA, LDHA, PARP1, SIRT5, BST1
Nortriptyline	1nM	Sigma-Aldrich	Lauterbach et al., 2013	Yes	SLC6A2, SLC6A4, HTR2A,

					HTR1A, HRH1, ADRA1A, ADRA1D, CHRM1, CHRM2, CHRM3, CHRM4, CHRM5, HTR2C, HTR6, ADRA1B, DRD2
Fingolimod phosphate	250nM	Santa Cruz Biotechnology	Pirhaji et al., 2016	No	--
Haloperidol	0.5nM	Cayman Chemical	Lauterbach et al., 2013	Yes	DRD2, DRD1, GRIN2B, HTR2A, DRD3, MCHR1, SLC18A2
Pizotifen	5uM	Sigma-Aldrich	Sarantos, Papanikolaou, Ellerby, and Hughes, 2012	Yes	CHRM1, CHRM2, CHRM3, HTR2A, HTR2B, HTR2C, HTR1A, HTR1B, HTR1D, HRH1, ADRA1A, ADRA1B, ADRA1D, ADRA2A, ADRA2B, ADRA2C
Cysteamine	250uM	Sigma-Aldrich	Bates, Tabrizi, and Jones, 2014	Yes	Cystine, SST, NPY2R
Sodium phenylbutyrate	100nM	Sigma-Aldrich	Bates, Tabrizi, and Jones, 2014	Yes	TYRB, NPR
Methylene blue	1nM	Sigma-Aldrich	Bates, Tabrizi, and Jones, 2014	Yes	GUCY1A2, NOS1
Rapamycin	1nM	Sigma-Aldrich	Bates, Tabrizi, and Jones, 2014	Yes	MTOR, FKBP1A, FGF2
Bezafibrate	100nM	Cayman Chemical	Chandra et al., 2016	Yes	PPARA, PPARD, PPARG, NR1I2, RXRA, RXRB, RXRG
(-)-Epigallocatechin gallate	100nM	Cayman Chemical	Zuccato, Valenza, and Cattaneo, 2010	No	AHR, DNMT1, DHFRL1
Creatine	500uM	Sigma-Aldrich	Bates, Tabrizi, and Jones, 2014	Yes	CKM, CKMT1A, CKB, CKMT2, SLC6A8, GAMT

Cystamine	250uM	Sigma-Aldrich	Bates, Tabrizi, and Jones, 2014	No	--
Desipramine	1uM	Sigma-Aldrich	Lauterbach et al., 2013	Yes	SLC6A2, SLC6A4, HTR2A, ADRB2, ADRB1, SMPD1, HRH1, ADRA1A, ADRA1B, ADRA1D, CHRM1, CHRM2, CHRM3, CHRM4, CHRM5, HTR1A, HTR2C, DRD2, ADRA2A, ADRA2B, ADRA2C
7,8-Dihydroxyflavone	100nM	Cayman Chemical	Jiang et al., 2013	No	--
Minocycline	10uM	Sigma-Aldrich	Bates, Tabrizi, and Jones, 2014	Yes	RPSL, RPSD, IL1B, ALOX5, MMP9, VEGFA, CASP1, CASP3, CYCS
Melatonin	10nM	Sigma-Aldrich	Lauterbach et al., 2013	Yes	MTNR1A, MTNR1B, ESR1, RORB, CALM1, MPO, EPX, CALR, ASMT, NQO2
Suberoylanilide hydroxamic acid	1nM	Sigma-Aldrich	Hockly et al., 2003	Yes	HDAC1, HDAC2, HDAC3, HDAC6, HDAC8, ACUC1
Curcumin	10nM	Sigma-Aldrich	Bates, Tabrizi, and Jones, 2014	Yes	PPARG, VDR, ABCC5, CBR1, GSTP1
Celastrol	1nM	Sigma-Aldrich	Wang, Gines, MacDonald, and Gusella, 2005	No	--
Fingolimod	1uM	Sigma-Aldrich	Di Pardo et al., 2014	Yes	S1PR5, HDAC1
Juglone	1nM	Sigma-Aldrich	Wang, Gines, MacDonald, and Gusella, 2005	No	--

Table S4. GO enrichment for the differentially expressed proteins affected by Group A compounds.

Related to Figure 3.

GO Term	Description	P-value	FDR-adjusted p-value	Enrichment score	Number of total proteins (#TotP)	#TotP in GO term	Number of Group A differential proteins (#DEP_A)	#DEP_A in GO term
GO:0030199	Collagen fibril organization	1.38 E-05	4.07E-02	2.48	6098	26	1703	18
GO:0043413	Macro-molecule glycosylation	1.07 E-05	4.21E-02	1.9	6098	68	1703	36

In Excel files:

Table S2. GO enrichment for the differentially expressed genes affected by Group A compounds.
Related to Figure 3.

Table S3. Pathway enrichment using IMPaLA for the differentially expressed metabolites affected by Group A compounds.
Related to Figure 3.

Table S5. GO enrichment for the differentially expressed genes affected by Group B compounds.
Related to Figure 3.

Table S6. Pathway enrichment using IMPaLA for the differentially expressed metabolites affected by Group B compounds.
Related to Figure 3.

Table S7. GO enrichment for the proteins in the Group A network.
Related to Figure 4.

Table S8. GO enrichment for the proteins in the Group B network.
Related to Figure 4.

Supplemental Methods

Transcription Factor Prediction

ChIP-Seq adapter sequences were trimmed from sequencing reads using Trimmomatic-0.36 and reads were aligned to the mm10 genome using Bowtie2 [1,2]. Reads were sorted and indexed, and mitochondrial DNA was removed using samtools-1.3 [3]. Peaks were called using MACS2 [4]. Motif analysis was used to predict transcription factors that could be regulating the differentially expressed genes. Motifs were annotated to the mm10 UCSC reference genome (http://genome.ucsc.edu/) using the CIS-BP database [5,6]. A hypergeometric test was used for each transcription factor to find those with motifs in regions intersecting ChIP-Seq peaks and within 2kb of differentially expression genes for a given condition. A Benjamini-Hochberg corrected p-value threshold of 0.05 was applied to assign significance to transcription factor predictions.

Metabolomics

Lipid Profiling

For lipid profiling, cells were resuspended in 50uL 60/35/5 acetonitrile/isopropanol/water (v/v/v) and 5uL was injected for LC/MS analysis. Please see Keckesova et al. and Smulan et al. for a detailed description of the LC/MS analysis [7,8]. Lipid identification and relative quantification was performed using LipidSearch (ThermoFisher Scientific / Mitsui Knowledge Industries). The identified lipids were subjected to quality control filtering and normalization by total signal [8].

Polar Metabolite Profiling

For polar metabolite profiling, cells were resuspended in 100uL water and 2uL was injected for LC/MS analysis. Please see Birsoy et al. and Chen at al. for a detailed description of the LC/MS analysis [9,10]. Untargeted analysis was performed using Progenesis CoMet (Nonlinear Dynamics) using the default settings. Features were filtered based on replicate injections and a dilution series of a pooled sample prepared by mixing equal aliquots of the biological samples. Specifically, the filtering criteria were CV < 0.4 across the four replicate injections and R > 0.9 across a four-point dilution

series (comprising 0.1X, 0.3X and 1X concentrations, and a double-volume injection). Features that were not lowest according to the Progenesis quantification in the blank water injection samples were discarded.

Proteomics

Sample Preparation

All solutions are reported as final concentrations. Lysis buffer (8 M Urea, 200 mM EPPS pH 8, Protease and Phosphatase inhibitors from Roche) was added to the vehicle and compound-treated cell pellets. The pellets were vortexed and sonicated to complete cell lysis. Protein concentration of the lysate was determined by micro-BCA assay (Pierce). Proteins were reduced with 5mM TCEP at room temperature for 15 minutes and alkylated with 10 mM Iodoacetamide at room temperature for 30 minutes in the dark. Proteins were precipitated using methanol and chloroform. Four volumes of methanol were added to the cell lysate, followed by one volume of chloroform, and three volumes of water. The mixture was vortexed and centrifuged to separate the chloroform phase from the aqueous phase. The precipitated protein was washed with one volume of ice-cold methanol. The washed precipitated protein was air dried. Precipitated protein was resuspended in 200 mM EPPS pH 8. Proteins were digested with LysC (1:50; enzyme:protein) overnight at room temperature and then further digested with trypsin (1:100; enzyme:protein) for another 8 hours at 37°C. Peptide concentration was quantified using the micro-BCA assay (Pierce). Peptide (100ug) from each condition was labeled with tandem mass tag (TMT6) reagents (1:4; peptide:TMT label) (Pierce) for 2 hours at room temperature. Modification of tyrosine residues with TMT was reversed by the addition of 5% hydroxylamine for 15 minutes at room temperature. The reaction was quenched with 0.5% TFA. Samples were combined at a 1:1:1:1:1:1 ratio, desalted by C18 solid-phase extraction (SPE, Sep-Pak, Waters), and dried by speedvac.

Phosphopeptide Enrichment

Phosphopeptides were enriched using the High-SelectTM Fe-NTA Phosphopeptide Enrichment Kit (ThermoFisher Scientific). Briefly, the combined TMT6 labeled peptides were resuspended in 200µl binding buffer and incubated with equilibrated resin for 30 minutes at room temperature. Unbound peptides were removed and saved for total protein analysis. Resin was washed and bound peptides were eluted with elution buffer. Eluted peptides were dried by speedvac, resuspended in 1% TFA, desalted by C18 SPE, and dried again. Peptides were resuspended and eluted into glass MS vials from a stage tip packed in-house with 3M Empore resin into two fractions at 20% and 70% ACN with 0.1% Formic acid. Eluted peptides were dried and resuspended in 5% Formic Acid, 5% ACN for MS analysis.

Peptide Fractionation

Peptide fractionation was performed by HPLC bRP. The unbound fraction from IMAC enrichment was dried by speedvac, resuspended in 1% TFA, and cleaned by C18 SPE. The desalted sample was dried by speedvac, resuspended in 5% ACN, 10mM ammonium bicarbonate pH8, and fractionated off-line by basic pH reversed-phase into 96 fractions. Separation was performed using a 50-minute linear gradient from 15% to 45% acetonitrile in 10mM ammonium bicarbonate pH 8 at a flow rate of 0.4mL/min over a 300 Extend C18 column (Agilent). Fractions were combined in checkerboard fashion into 24 samples and dried by speedvac.

Liquid Chromatography-MS3 Spectrometry

Of the 24 final fractions from the basic reverse phase, 12 fractions were analyzed with LC-MS3 on an Orbitrap Fusion mass spectrometer (ThermoFisher Scientific) equipped with a Proxeon Easy nLC 1000 for online sample handling and peptide separations. Approximately 5 µg of peptide resuspended in 5% formic acid with 5% acetonitrile was loaded onto a 100 µm inner diameter fused-silica micro capillary with a needle tip pulled to an internal diameter less than 5 µm. The column was packed in-house to a length of 35 cm with a C18 reverse phase resin (GP118 resin 1.8 µm, 120 Å, Sepax Technologies). The peptides were separated using a 180-minute linear gradient from 3% to 25% buffer B (100% ACN + 0.125% formic acid) equilibrated with buffer A (3%

ACN + 0.125% formic acid) at a flow rate of 600 nL/min across the column. The scan sequence for the Fusion Orbitrap began with an MS1 spectrum (Orbitrap analysis, resolution 120,000, 350−1500 m/z scan range, AGC target 4 × 105, maximum injection time 50 ms, dynamic exclusion of 120 seconds). The “Top10” precursors were selected for MS2 analysis, which consisted of CID (quadrupole isolation set at 0.7 Da) and ion trap analysis, AGC 1 × 104, NCE 35, maximum injection time 120 ms). The top ten precursors from each MS2 scan were selected for MS3 analysis (synchronous precursor selection), in which precursors were fragmented by HCD prior to Orbitrap analysis (NCE 65, max AGC 1 × 105, maximum injection time 150 ms, isolation window 2 Da, resolution 50,000.

Phosphopeptide Data Collection

Phosphopeptide samples were analyzed with LC-MS3 on an Orbitrap Lumos mass spectrometer (ThermoFisher Scientific) equipped with a Proxeon Easy nLC 1000 for online sample handling and peptide separations. Total peptide was resuspended in 5% formic acid + 5% acetonitrile was loaded onto a 100 µm inner diameter fused-silica micro capillary with a needle tip pulled to an internal diameter less than 5 µm. The column was packed in-house to a length of 35 cm with a C18 reverse phase resin (GP118 resin 1.8 µm, 120 Å, Sepax Technologies). The peptides were separated using a 180-minute linear gradient from 3% to 25% buffer B (100% ACN + 0.125% formic acid) equilibrated with buffer A (3% ACN + 0.125% formic acid) at a flow rate of 600 nL/min across the column. The scan sequence for the Fusion Orbitrap began with an MS1 spectrum (Orbitrap analysis, resolution 120,000, 400−1400 m/z scan range, AGC target 1 × 106, maximum injection time 100 ms, dynamic exclusion of 120 seconds). The “Top10” precursors were selected for MS2 analysis, which consisted of CID (quadrupole isolation set at 0.5 Da) and ion trap analysis, AGC 2 × 104, NCE 35, maximum injection time 60 ms). The top ten precursors from each MS2 scan were selected for MS3 analysis (synchronous precursor selection), in which precursors were fragmented by HCD prior to Orbitrap analysis (NCE 65, max AGC 2 × 105, maximum injection time 300 ms, isolation window 2 Da, resolution 50,000.

LC-MS3 Data Processing and Analysis

A suite of in-house software tools was used for .RAW file processing and controlling peptide and protein level false discovery rates, assembling proteins from peptides, and protein quantification from peptides [11,12]. MS/MS spectra were searched against a Uniprot mouse database with both the forward and reverse sequences. Database search criteria are as follows: tryptic with two missed cleavages, a precursor mass tolerance of 50 ppm, fragment ion mass tolerance of 1.0 Da, static alkylation of cysteine (57.02146 Da), static TMT labeling of lysine residues and N-termini of peptides (229.162932 Da), and variable oxidation of methionine (15.99491 Da). TMT reporter ion intensities were measured using a 0.003 Da window around the theoretical m/z for each reporter ion in the MS3 scan. Peptide spectral matches with poor quality MS3 spectra were excluded from quantitation (<100 summed signal-to-noise across 6 channels and <0.5 precursor isolation specificity). Phosphopeptide searches included variable phosphorylation on serine, threonine, and tyrosine residues (79.96633 Da). Phosphorylation site localization was scored with ModScore. Phosphorylation sites with summed signal-to-noise <100 across all 6 channels and/or <0.5 precursor isolation specificity were excluded from quantitation.

Pathway Enrichment

Enrichment analyses of the differential genes, differential proteins, and network proteins were performed using GOrilla with a background set of all genes measured, all proteins measured, or all proteins present in the interactome, respectively [13]. Enrichment analyses of the differential metabolites were performed using IMPaLA with a background set of all metabolites measured [14].

t-SNE Analysis

t-SNE was used to display the transcriptomic and metabolomic data as two-dimensional projections. The inputs were matrices including gene, lipid, or polar metabolite quantifications for each sample and perplexities were set to 15, 14, and 14, respectively. t-SNE analysis was performed in R using the Rtsne package [15].

PCA Analysis

Because the number of samples in the proteomic data was lower than in the other omics data types, t-SNE analysis was not applicable. Instead, we displayed the protein and phosphosite data as three-dimensional PCA plots using the stats and rgl packages in R [16,17].

Network Visualization

Networks were visualized in Cytoscape [18]. In each network, the nodes are proteins, phosphosites, transcription factors, or metabolites. The proteomic data are mapped onto proteins and phosphosites. The integration of the RNA-Seq and ChIP-Seq data provided transcription factor predictions. The metabolite data is shown as metabolite peaks connected to m/z-matched known metabolites. The edges represent the physical interactions between the molecules. Bigger nodes are more robust, as determined by the PCSF randomizations. The red and blue colors indicate the log2 fold change, as determined by the omics data.

Dendrogram Clustering

Using the controls and the four compounds in Groups A and B analyzed with all of the omics data, a distance matrix was calculated for each data type using the Euclidean distance measure in the stats package in R [17]. Dendrograms were created using the distance matrices for each data type using the hclust function with the Ward clustering method in the stats package in R [17].

Calculating Structural Similarities

Identifiers for each compound were uploaded to ChemMine tools and the “Similarity Workbench” feature was used to compare each pair of compounds. The tool calculates atom pair and reports maximum common substructure (MCS) scores with the Tanimoto coefficient as the similarity measure [19].

Calculating Connectivity Similarities

The L1000 connectivity scores between pairs of compounds were assessed using the "Touchstone" analysis tool as part of the Connectivity Map [20,21]. Only eight of the 30 compounds profiled were part of the Connectivity Map dataset.

References

1. Bolger, A. M., Lohse, M. & Usadel, B. Trimmomatic: A flexible trimmer for Illumina sequence data. *Bioinformatics* **30**, 2114–20 (2014).

2. Langmead, B. & Salzberg, S. L. Fast gapped-read alignment with Bowtie 2. *Nat. Methods* **9**, 357–9 (2012).

3. Li, H. *et al.* The Sequence Alignment/Map format and SAMtools. *Bioinformatics* **25**, 2078–9 (2009).

4. Zhang, Y. *et al.* Model-based Analysis of ChIP-Seq (MACS). *Genome Biol.* **9**, R137 (2008).

5. Waterston, R. H. *et al.* Initial sequencing and comparative analysis of the mouse genome. *Nature* **420**, 520–62 (2002).

6. Weirauch, M. T. *et al.* Determination and Inference of Eukaryotic Transcription Factor Sequence Specificity. *Cell* **158**, 1431–43 (2014).

7. Smulan, L. J. *et al.* Cholesterol-Independent SREBP-1 Maturation Is Linked to ARF1 Inactivation. *Cell Rep.* **16**, 9–18 (2016).

8. Keckesova, Z. *et al.* LACTB is a tumour suppressor that modulates lipid metabolism and cell state. *Nature* **543**, 681–6 (2017).

9. Chen, W. W., Freinkman, E., Wang, T., Birsoy, K. & Sabatini, D. M. Absolute Quantification of Matrix Metabolites Reveals the Dynamics of Mitochondrial Metabolism. *Cell* **166**, 1324–37.e11 (2016).

10. Birsoy, K. *et al.* An Essential Role of the Mitochondrial Electron Transport Chain in Cell Proliferation Is to Enable Aspartate Synthesis. *Cell* **162**, 540–51 (2015).

11. McAlister, G. C. *et al.* MultiNotch MS3 enables accurate, sensitive, and

multiplexed detection of differential expression across cancer cell line proteomes. *Anal. Chem.* **86**, 7150–8 (2014).

12. Weekes, M. P. *et al.* Quantitative temporal viromics: An approach to investigate host-pathogen interaction. *Cell* **157**, 1460–72 (2014).

13. Eden, E., Navon, R., Steinfeld, I., Lipson, D. & Yakhini, Z. GOrilla: a tool for discovery and visualization of enriched GO terms in ranked gene lists. *BMC Bioinformatics* **10**, 48 (2009).

14. Kamburov, A., Cavill, R., Ebbels, T. M. D., Herwig, R. & Keun, H. C. Integrated pathway-level analysis of transcriptomics and metabolomics data with IMPaLA. *Bioinformatics* **27**, 2917–18 (2011).

15. Krijthe, J. H. T-Distributed Stochastic Neighbor Embedding using Barnes-Hut. *T-Distributed Stochastic Neighbor Embedding using Barnes-Hut* (2015). doi:10.5430/rwe.v8n2p49

16. Adler, D., Nenadić, O. & Zucchini, W. RGL: A R-library for 3D visualization with OpenGL. in *Proceedings of the 35th Symposium of the Interface: Computing Science and Statistics* (2003).

17. R Core Team. R: A language and environment for statistical computing. http://www.R-project.org/. *R Foundation for Statistical Computing, Vienna, Austria* (2017).

18. Shannon, P. *et al.* Cytoscape: A software Environment for integrated models of biomolecular interaction networks. *Genome Res.* **13**, 2498–504 (2003).

19. Backman, T. W. H., Cao, Y. & Girke, T. ChemMine tools: An online service for analyzing and clustering small molecules. *Nucleic Acids Res.* **39**, W486-91 (2011).

20. Lamb, J. *et al.* The connectivity map: Using gene-expression signatures to connect small molecules, genes, and disease. *Science (80-.).* **313**, 1929–35 (2006).

21. Subramanian, A. *et al.* A Next Generation Connectivity Map: L1000 Platform and the First 1,000,000 Profiles. *Cell* **171**, 1437–52.e17 (2017).
